# Complete Congenital Absence of Left Pericardium: A Case Report

**DOI:** 10.7759/cureus.92125

**Published:** 2025-09-12

**Authors:** Shreya Gupta, Dinesh kumar

**Affiliations:** 1 Department of Cardiology, Atal Bihari Vajpayee Institute of Medical Sciences (ABVIMS) and Dr. Ram Manohar Lohia (RML) Hospital, Delhi, IND; 2 Department of Cardiology, Saroj Super Speciality Hospital, Delhi, IND

**Keywords:** benign, congenital absence of pericardium, developmental defect, pericardial disease, pericardium

## Abstract

Congenital absence of the pericardium (CAP) is a rare anomaly, often silent and easily mistaken for other cardiac conditions. In this case report, a 72-year-old man without cardiovascular risk factors presented with intermittent, non-exertional left-sided chest discomfort over two weeks. His ECG showed absent R-wave progression, raising suspicion of coronary artery disease. Transthoracic echocardiography revealed a poorly defined cardiac silhouette with a leftward shift. The stress echo was negative for inducible ischemia. A chest X-ray was done to look into the cause of the displaced apex and showed loss of the right heart border, an elongated left cardiac contour, and normal lungs with a central trachea. The CT confirmed the complete absence of the left pericardium. No high-risk features of cardiac herniation were observed. Given the benign nature of complete unilateral pericardial absence and absence of complications, the patient was managed conservatively and reassured for his complaints. This case report highlights that recognition of characteristic imaging signs, especially on chest X-ray and CT, is crucial for distinguishing pericardial agenesis from other cardiothoracic conditions. Awareness of this rare entity aids in preventing unnecessary interventions and ensuring optimal patient care.

## Introduction

Congenital absence of the pericardium (CAP) is a rare cardiac malformation, with approximately 500 cases reported to date [1]. It is often asymptomatic and most often discovered as an incidental finding [2]. This is usually due to the defective development of the pleuropericardial membrane. It can present as the partial or complete absence of the pericardium, with the latter being a benign condition. Due to its rarity and atypical presentation, CAP is often misdiagnosed or diagnosed late. This report illustrates the case of an elderly patient referred for imaging in view of atypical chest pain with ECG changes. Initial evaluation raised suspicion for coronary artery disease, but further imaging revealed a complete absence of the left pericardium.

## Case presentation

**Figure 1:**
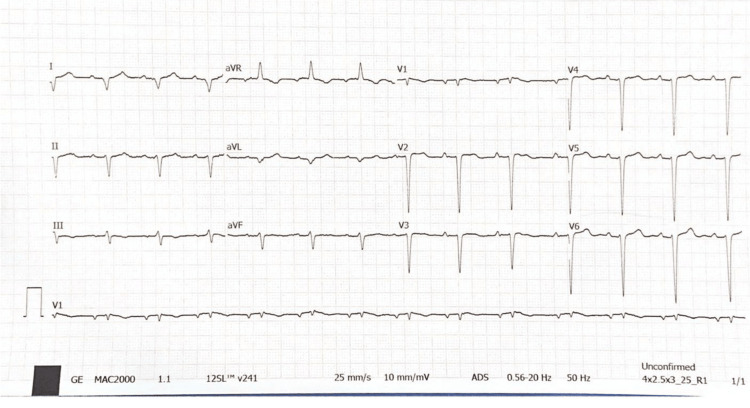
The ECG demonstrated sinus rhythm with marked right axis deviation and poor R-wave progression.

A 72-year-old male, a driver by occupation, with no known comorbidities, consulted medical help in view of on-and-off chest pain for two weeks. The pain was on the left side of the chest, which radiated to his left shoulder. It was non-exertional and unrelated to meals. Pedal edema, fatigue, or exertional dyspnea was not present. There was no history of substance abuse. The vitals of the patient were normal. Cardiovascular examination only revealed a displaced apex. Routine blood tests, including cardiac enzymes, were also normal. The ECG showed sinus rhythm and absent R wave progression (Figure 1).

**Figure 2:**
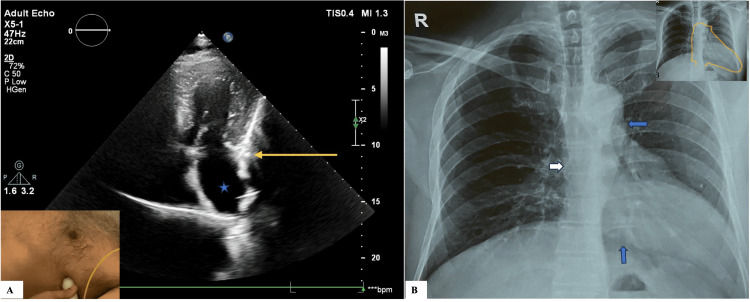
Echocardiography and X-ray radiography imaging A: A modified apical four-chamber view was obtained in the supine position with the transducer positioned near the posterior axillary line (box on bottom left corner). Elongated atria (star) with a widened ventricle demonstrating a classic ‘tear drop’ appearance were observed. The yellow arrow points to the abnormal sharp angulation between the atria and the ventricle. B: Chest radiograph demonstrating leftward cardiac displacement with obscuration of the right heart border (white arrow) and straightening of the left cardiac margin (‘Snoopy sign’ seen in the image on the top right corner). Also, a lucent area is seen between the aorta and pulmonary artery and between the left hemidiaphragm and the base of the heart (blue arrow).

Given the old age of the patient, atypical chest pain, and abnormal ECG, the possibility of coronary artery disease with old anteroseptal MI was kept. Echocardiography was ordered, and it revealed a poor echocardiographic window and a leftward-displaced heart. The modified apical four-chamber view (Figure 2A) showed elongated atria with widened ventricles, mild concentric left ventricular (LV) hypertrophy with type 1 LV diastolic dysfunction, and an ejection fraction (EF) of 62% with no regional wall motion abnormality. The stress echo was negative for inducible ischemia. Given the displaced apex found both on examination and echo, a chest X-ray was done, which showed a normal bony structure, including the spine, central trachea, and normal lung parenchyma, ruling out non-cardiac causes for the displaced apex. Subtle radiological signs, including leftward cardiac displacement, elongation with straightening of the left cardiac margin, and obscuration of the right heart border, were suggestive of underlying cardiac pathology. Additionally, an area of radiolucency was noted between the left hemidiaphragm and the heart’s base and also between the aorta and pulmonary artery (Figure 2B). 

**Figure 3:**
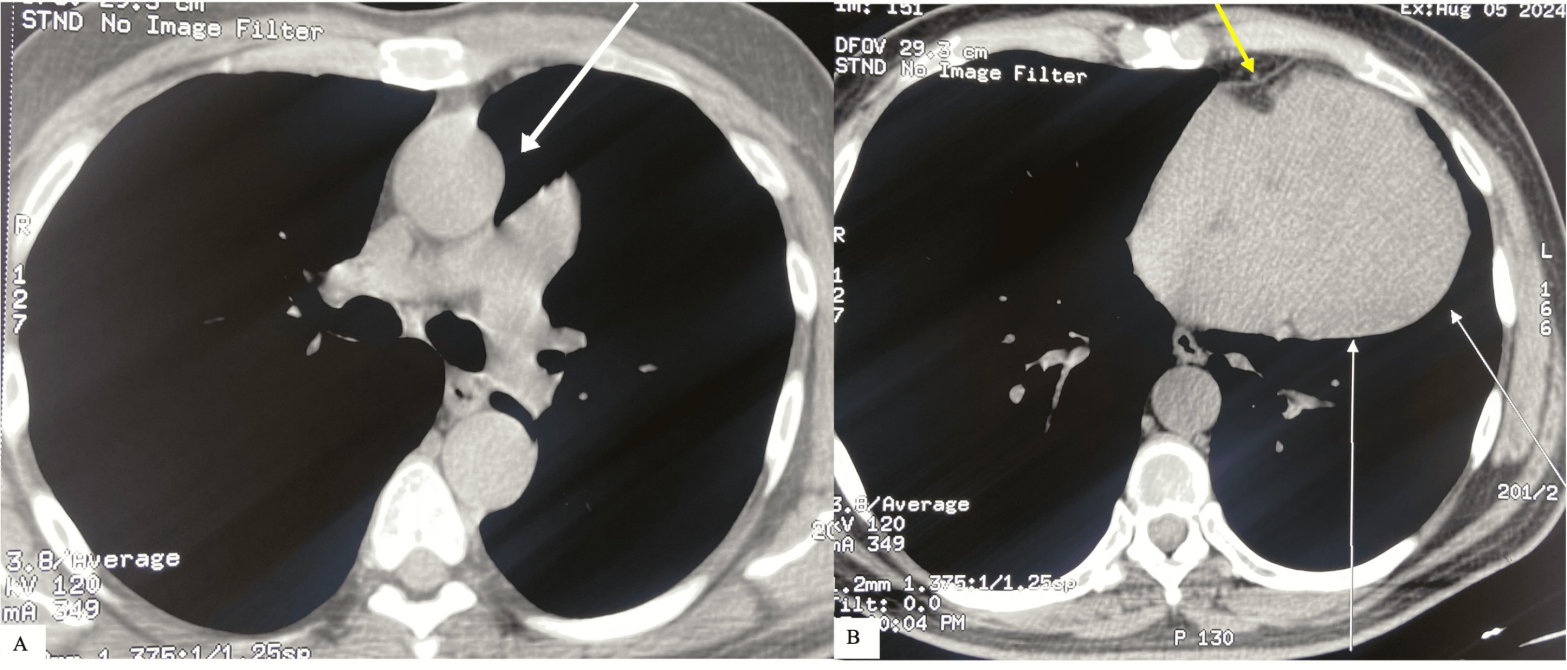
Cardiac CT axial frame images A: Demonstrates interposition of lung tissue between the aortic root and main pulmonary artery (arrow); B: Depicts complete absence of the pericardium along the left ventricular border (white arrows), with pericardium preserved only at the acute margin of the heart (yellow arrow). Also, note the leftward displacement of the heart into the hemithorax.

For confirmation, a chest CT scan was performed, which revealed a levo-positioned heart with a posteriorly oriented apex, along with complete absence of the left pericardium (Figure 3B). Additionally, interposed lung tissue was noted between the aorta and pulmonary artery (Figure 3A). Based on these CT findings, a diagnosis of congenital complete absence of the left pericardium was established. As there were no complications, the patient was reassured of his complaints and advised to follow up.

## Discussion

Congenital absence of the pericardium is a rare embryonic anomaly first described in 1793 by Mathew Baillie. As of 2023, only 500 cases have been reported, the reason being that most cases are asymptomatic or have an atypical presentation. Embryologically, failure of fusion of pleuropericardial folds, which usually occurs by the end of the fifth week of gestation, leads to CAP. Multiple theories have been postulated for the same. One theory states that there is differential growth of the heart and pericardium, due to which the heart stretches the enveloping pericardium, leading to the failure of the fusion of the pleuropericardial membrane, leading to CAP. Another widely accepted theory is premature atrophy of the left common cardinal vein (duct of Cuvier), which supplies the pleuropericardial folds, leading to the persistence of the embryonic pleuropericardial foramen, leading to CAP. This theory also explains why left pericardial defects are more common, since the right side of the Cuvier develops into the SVC, ensuring the closure of the right pleuropericardial folds. According to other theories, a tear in the developing pericardial membrane may be responsible [3,4]. 

The CAP may manifest as a complete defect or a foramen-type partial defect of one or both sides. Left-sided CAP is the most common type (70%), followed by complete (23.1%) and right-sided (5%) [1]. Although CAP is often detected incidentally, patients may present with a wide spectrum of manifestations ranging from acute life-threatening events to benign chronic cardiac symptoms. In contrast to individuals with complete bilateral or left-sided pericardial absence, those with partial defects tend to be more symptomatic and are at higher risk for complications. Partial absence of the left pericardium represents the most serious variant, as it may permit herniation of the left atrial appendage, the entire left atrium, or both ventricles, potentially resulting in cardiac strangulation or coronary artery compression [2,5]. Complete absence of the pericardium is the variant least commonly associated with complications. Patients typically present with atypical chest pain, often sharp and stabbing on the left side, attributable to tension from pleuropericardial adhesions, absence of pericardial cushioning, or excessive torsion and strain on the great vessels. The most extensive systematic review involving 247 cases (1977-2023) of CAP done by Peir et al. showed chest pain and dyspnea as the most common presentation [1]. Other symptoms like trepopnea, an uncomfortable pounding sensation, and shifting heart sensations can also be seen. Acute coronary syndrome, hilar lymphadenopathy, and lung and cardiac tumors can all have symptoms that are similar. 

Concomitant congenital cardiac abnormalities have been documented in 20% to 30% of patients, encompassing atrial septal defects, patent ductus arteriosus, tetralogy of Fallot, bicuspid aortic valve, and mitral valve stenosis or prolapse [1,6]. Physical examination is usually not characteristic or helpful in making a diagnosis. Patients may have apical displacement of the point of maximal impulse if they have no left pericardium at all. The base of an abnormally mobile heart may have a systolic ejection murmur near the left sternal border as a result of turbulence caused by several mechanical abnormalities.

The ECG usually shows poor R wave progression (leftward displacement of the precordial transition zone), right axis deviation, and incomplete right bundle branch block. Other changes seen are peaked P waves, postural changes in the QRS vector, electrical alternans, sinus bradycardia, and marked sinus arrhythmia [7]. On chest X-rays, patients with complete absence of the left pericardium show obscuration of the right heart border (silhouetted by the spine), radiolucent bands of lung tissue interposed between the aortic knob and main pulmonary artery as well as between the left hemidiaphragm and the cardiac base, leftward and posterior displacement of the cardiac apex, and elongation with straightening of the left heart border ('Snoopy sign' ) [2,8,9]. Since this condition does not result in tracheal deviation despite all the cardiac rotations observed, the clinicians should be prompted to take this condition into consideration when there is a displaced apex with a central trachea.

Although transthoracic echocardiography (TTE) may demonstrate characteristic features, these findings can often be overlooked even by experienced interpreters because standard views do not apply here. The patient may receive a false diagnosis of right ventricular dilatation because of the left shift of the heart on the traditional left parasternal view. In the apical window obtained with the patient in the left lateral decubitus position, the heart appeared markedly displaced laterally with evidence of atrial compression. Modified views are used for better visualization. Other findings include abnormal sharp atrial-ventricular angle (A4C), elongated atria, widened ventricles ('tear-drop appearance'), exaggerated mobility of the heart (cardioptosis), and paradoxical septal motion in systole [2,7,9,10].

Cardiac CT/MRI confirms the diagnosis. The presence of lung tissue between the aorta and pulmonary artery or between the diaphragm and the base of the heart, levorotation of the heart, absence of the pericardial layer, and the presence of a subepicardial myocardial crease, suggestive of external compression from a partial pericardial defect, are signature findings. An ECG-gated cardiac CT or cardiovascular magnetic resonance (CMR) allows high-resolution structural assessment. Cine CMR further offers dynamic evaluation of cardiac function, which is particularly valuable in partial pericardial absence for detecting herniation through the band-like foramen formed by the defect [2,7].

Management

Given the rarity of this condition, large controlled studies are lacking, and management recommendations are primarily derived from case reports and observational data. In most patients, the condition is clinically silent and requires no intervention. Surgical treatment is generally reserved for partial defects in symptomatic individuals or in those with high-risk features such as cardiac herniation or coronary artery ischemia. Surgical alternatives include patch closure of the defect, pericardiectomy, or pericardioplasty (dilation of the pericardial defect to prevent strangulation) [7].

## Conclusions

Although congenital pericardial absence is an uncommon cardiac condition, it could be mistaken for other cardiac conditions like ischemic heart disease, extracardiac mass lesions, etc. Baseline testing for any patient with suspected cardiac or pulmonary-related symptoms includes ECG, chest X-ray, and TTE. Therefore, recognition of characteristic imaging signs is crucial for distinguishing pericardial agenesis from other cardiothoracic conditions. If suggestive of CAP, a definitive diagnosis is based on cardiac CT or CMR. In asymptomatic or low-risk cases of complete pericardial absence, conservative management with regular monitoring is sufficient. Awareness of this rare entity aids in preventing unnecessary interventions and ensures optimal patient care.
